# Continuum beliefs of mental illness: a systematic review of measures

**DOI:** 10.1007/s00127-022-02345-4

**Published:** 2022-08-05

**Authors:** S. Tomczyk, S. Schlick, T. Gansler, T. McLaren, H. Muehlan, L.-J. Peter, G. Schomerus, S. Schmidt

**Affiliations:** 1 Department Health and Prevention, Institute of Psychology, University of Greifswald, Robert-Blum-Straße 13, 17489 Greifswald, Germany; 2 Department of Psychiatry and Psychotherapy, Medical Faculty, Leipzig University, Leipzig, Germany; 3 Department of Psychiatry and Psychotherapy, University of Leipzig Medical Center, Leipzig, Germany

**Keywords:** Mental health, Public health, Systematic review, Stereotyping, Continuum, Assessment

## Abstract

**Purpose:**

The continuum of mental health/illness has been subject to scientific debate for decades. While current research indicates that continuum belief interventions can reduce mental health stigma and improve treatment seeking in affected populations, no study has yet systematically examined measures of continuum beliefs.

**Methods:**

This preregistered systematic review summarizes measures of continuum beliefs. Following the PRISMA statement, three scientific databases (PubMed, PsycInfo and PsycArticles via EBSCOhost, Web of Science) are searched, instruments are described and discussed regarding their scope, and methodological quality.

**Results:**

Overall, 7351 records were identified, with 35 studies reporting relevant findings on 11 measures. Most studies examined general population samples and used vignette-based measures. Schizophrenia and depression were most commonly examined, few studies focused on dementia, ADHD, OCD, eating disorders, and problematic alcohol use, or compared continuum beliefs across disorders. Validity was very good for most measures, but reliability was rarely tested. Measures mostly assessed beliefs in the normality of mental health symptoms or the normality of persons with such symptoms but rarely nosological aspects (i.e., categorical v continuous conceptualization of mental disorders).

**Conclusions:**

Current research provides psychometrically sound instruments to examine continuum beliefs for a variety of mental disorders. While studies suggest utility for general population samples and mental health professionals, more research is necessary to corroborate findings, for instance, regarding age (e.g., in adolescents), gender, or type of mental disorder. Future research should also compare self-report ratings, and vignette-based measures, include measures of nosological concepts to fully grasp the continuum concept of mental illness.

**Preregistration:**

PROSPERO: CRD42019123606.

## Introduction

The nosological concept of mental disorders has been subject to long-standing discussions. To date, there is no undisputable consensus on their categorical or dimensional nature, although developments of the DSM 5 [1] as well as comprehensive literature seem to favor continuous measures of psychopathology which furthers a dimensional understanding [2, 3]. Schizophrenia, for example, is described along the proneness–persistence–impairment continuum describing psychotic and subsyndromal experiences among the general population with only a small proportion reporting persistent symptoms that may lead to an impairment [4, 5]. This concept has implications for prevention, diagnosis and treatment, as it informs researchers, policymakers and practitioners alike. For example, a continuum model of schizophrenia emphasizes the need for selective prevention in at-risk groups [6], and identifies subgroups with persistent symptoms for personalized treatment purposes. It also points to groups with subsyndromal experiences as target groups for early prevention [7]. A categorical understanding of schizophrenia, on the other hand, facilitates stigmatizing attitudes, because it allows a clear distinction of social groups, that is people with and without schizophrenia [8]. It should be noted, however, that other researchers criticize such a continuum model from a methodological perspective [9, 10]. Linscott and van Os [9], for example, point to methodological flaws and challenges of the conception of continua that might overshadow categorically derived findings, such as latent classes. A similar debate between categorical and continuous conceptualizations can be seen for eating disorders [11–13], obsessive–compulsive disorder [14], generalized anxiety disorder [15], depression [16, 17], and at-risk substance use [18, 19] or gambling [20]. This debate is not limited to the scientific community but it also affects patients and the public. Previous research shows that the public perception of mental illness as a categorical construct is connected to public stigma [21, 22] and mental health stigma is recognized as a barrier to treatment seeking [23–29]. It is also linked to negative psychosocial outcomes, for example, lower self-esteem and self-efficacy and poor quality of life [30–34]. Conversely, a continuum model of mental illness is related to more positive mental health outcomes [35], and lower stigmatizing attitudes. Therefore, promoting continuum beliefs to the public might be a promising approach to reducing public stigma [36].

In this manner, Angermeyer and Schulze [21] describe two core strategies of public communication in line with either categorical beliefs (i.e., medicalization) or continuum beliefs (i.e., normalization). The first strategy encompasses medical treatments of individuals with distinct disorders, such as schizophrenia, and is more prominent among medical professionals and connected to biomedical causal beliefs of mental disorders [37–39]. The second strategy sees psychiatric symptoms as a normal experience but connects mental disorders to an increased level of stress and insufficient coping resources. It is more prominent among non-medical health care workers as well as support groups, and it is more strongly connected to psychosocial causal beliefs [37, 38]. In spite of their potential for public mental health and social psychiatry, for instance, by reducing stigmatizing attitudes and thus lowering the barrier to entry into treatment no study has systematically reviewed and summarized measures for continuum beliefs regarding mental health and mental illness, which makes it difficult to assess their validity and utility. For instance, an experienced-based measure might be more valid for clinical samples but less applicable to general population samples, whereas a vignette-based measure might be more applicable but also more strongly affected by bias (e.g., gender bias in case of gendered vignettes). Therefore, this systematic review aims to review and assess previously utilized measures for continuum beliefs to harmonize research efforts and answer the following questions.

1. What are the characteristics of existing continuum belief instruments (e.g., country of origin, setting/target group, examined disorders, mode of administration)?
2. What are the psychometric properties of continuum belief measures?
3. Which areas of the continuum of mental health and mental illness are covered by continuum belief measures?

## Method

This systematic review was conducted in accordance with the Preferred Reporting Items for Systematic Reviews and Meta-Analyses (PRISMA) Statement [40] and is registered with the PROSPERO registry (https://www.crd.york.ac.uk/prospero; CRD42019123606). Three scientific databases (PubMed, PsycInfo and PsycArticles via EBSCOhost, Web of Science) were searched for peer-reviewed articles on continuum beliefs that were published before June 2022. The search was performed in line with a review and meta-analysis on the association between continuum beliefs and mental health stigma [36]; therefore, initial database search and abstract and title screening was similar in this study, but eligibility criteria differed between studies. Search terms comprised continuum AND stigma AND mental health OR mental illness, search strategies are presented in Peter et al. [36]. In addition, reference lists of included studies were checked to identify additional eligible studies.

### Eligibility criteria

Eligibility criteria were described in accordance with the PICO process [41]:

Population: Human beings from the general population without any age restrictions.

Intervention: Studies that investigate continuum beliefs were included, either as observational or interventional studies. Continuum beliefs refer to the nosological concept of mental illness, either as a general, transdiagnostic concept of continuity of mental illness/mental health problems or as a specific concept for distinct mental disorders. Other forms of continua, such as the continuum of care [42] or the dual-continua model of mental health and mental illness [43–45], were not included, because they represent broader concepts within psychiatric and psychological research regarding health care structures as well as psychological functioning, which transcend the current research question that focuses on the conceptualization of mental disorders.

Comparison: Experimental as well as observational quantitative studies were included; therefore, there was no restriction regarding a potential control group.

Outcome: Studies should measure continuum beliefs, either as a predictor, an intermediary variable, or as an outcome.

Studies were not limited to a particular design (e.g., experimental studies or observational cohort studies) or method (e.g., quantitative data assessment). Finally, the search was limited to studies published in English, German, French, or Polish. Titles and abstracts of identified studies were screened by the first and second author and full texts were obtained of potentially relevant studies. Full texts were then screened against eligibility criteria independently by the first and second author. Differences were discussed with the third author and solved by mutual agreement to include or exclude studies.

### Data extraction, synthesis, and analysis

The first and second author independently extracted data on authors, date of publication, study design, sample, measures and psychometric properties (if reported in the original studies). The first and third author then independently rated dimensions of methodological quality and psychometric properties of the measures following the reporting guidelines proposed by Bennett et al. [46] to compare measures. The following dimensions were examined: readability (availability and length of the measure), cultural translation (availability in multiple (target) languages), respondent burden (over/under 60 items), content validity (theoretical foundation and expert consultation), criterion validity (correlation with external criteria), construct validity (correlation with related/non-related constructs), internal consistency (Cronbach’s alpha below/above 0.70), inter-rater reliability (agreement between different raters), intra-rater reliability (agreement within one rater), test–retest reliability (significant test–retest correlation across at least two timepoints), floor or ceiling effects, and responsiveness (successful manipulation check). The definitions are also listed in the table notes of Table 3, but a concise definition of these aspects can be found elsewhere [47]. Differences in ratings or extracted information were discussed and solved with the second author. The narrative synthesis reports identified measures of continuum beliefs, their assessment method, their content as well as a rating of their methodological quality. For each study, design, sample size and composition, and country of origin are also reported.

## Results

**Fig. 1:**
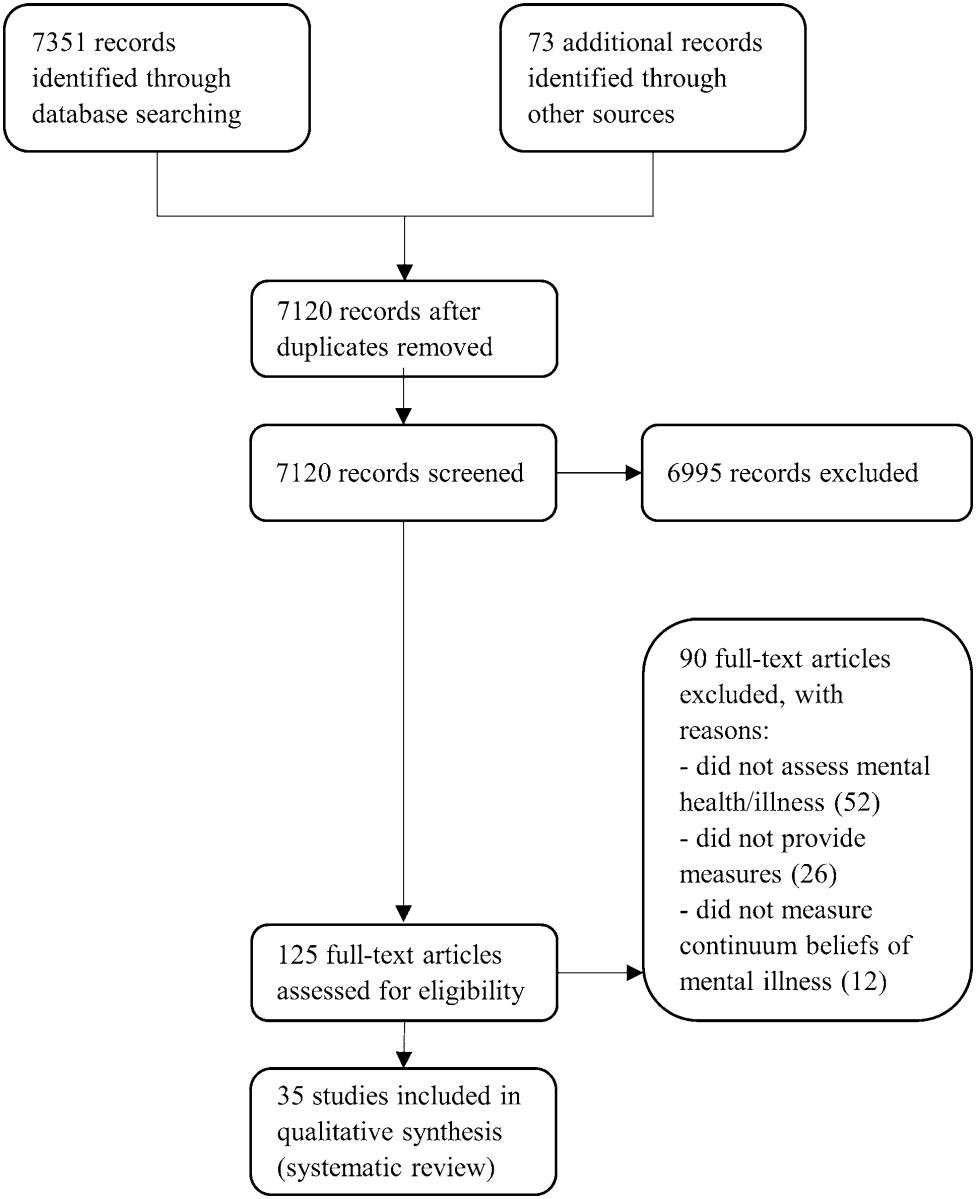
PRISMA flow diagram

The initial database search resulted in 7351 records (PubMed: 3197, Web of Science: 2209, EBSCOhost: 1945), with 73 records being additionally identified from reference lists of potentially relevant studies. After removing duplicates, 7120 records remained. A screening of titles and abstracts lead to an exclusion of 6995 records. Finally, 125 full texts were assessed for eligibility, wherefrom 90 studies were excluded, leading to a sample of 35 studies for the synthesis (see Fig. 1).

The excluded studies did not assess mental health/illness but other aspects, such as the continuum of care; they did not provide measures (e.g., editorials or theoretical work) or they were based on other concepts of a continuum such as the dual continua model [43, 45] that refer to psychological functioning (i.e., the intersection of mental wellbeing and mental health/illness) rather than nosological concepts of mental health/illness.

### Study description

The included studies [22, 48–81] investigated continuum beliefs regarding multiple mental disorders (more than one disorder per study in 15 out of 35 studies). Most studies were conducted in Germany (*n* = 14), followed by the United States of America (*n* = 10), Australia, Canada, France, and Singapore (*n* = 2) as well as United Kingdom, Ireland and the Netherlands (*n* = 1). An overview of included studies is given in Table 1.

**Table 1:** Overview of included studies measuring continuum beliefs (*n* = 35)

No	Study	Design	Sample	Population	Country	Measure	No. of items	Examined disorders	Method	Response scale
1	Angermeyer et al. (2015)	Cross-sectional (online)	*n* = 1600; 16–65 years; 50% male	General population (representative)	France	Belief in a continuum of symptom experience	1	Depression; schizophrenia	Vignette	Likert (1–5)
2	Bahlmann et al. (2015)	Cross-sectional (same as no. 20)	*n* = 3642; > 18 years	General population (representative)	Germany	Belief in a continuum of symptom experience	1	Depression; schizophrenia; alcohol use disorder	Vignette	Likert (1–5)
3	Buckwitz et al. (2021)	Online experiment	*n* = 478; mean age = 34.1 years; 59% male	MTurk sample	USA	Belief in a continuum of symptom experience	3	Depression	Rating	Likert (1–5)
4	Buckwitz et al. (2022)	Online experiment (same as no. 3)	*n* = 304; mean age = 34.1 years; 59% male	MTurk sample	USA	Belief in a continuum of symptom experience	3	Depression	Rating	Likert (1–5)
5	Cassidy et al. (2020)	Online experiment	*n* = 398; 18–75 years; mean age = 36.76 years; 50.3% male	MTurk sample	USA	Belief in a continuum of symptom experience	4	Bipolar disorder	Vignette	Likert (1–5)
6	Cole et al. (2019)	Online experiment	*n* = 178; mean age = 38.01 years; 35.4% male	MTurk sample	USA	Continuum and categorical beliefs	1	OCD	Vignette	Likert (0–4)
7	Corrigan et al. (2016)	Online experiment	*n* = 598; mean age = 35.6 years; 48.3% male	MTurk sample	USA	CBQ	16	Schizophrenia	Vignette	Likert (1–6)
8	Dolphin et al. (2017)	Online experiment	*n* = 156; mean age = 16.25 years; 48.7% male	Students	Ireland	Agreement with continuum scale	1	Depression	Vignette	Likert (1–6)
9	Fernandez et al. (2022a)	Cross-sectional (online)	*n* = 193; mean age = 17.5 years; 21% male	Adolescents (community sample)	Australia	Continuity beliefs; fundamental differences	4	Depression; schizophrenia	Vignette	Likert (1–7)
10	Fernandez et al. (2022b)	Cross-sectional (online)	*n* = 271; mean age = 31.7 years; 52% male	General population	Australia	Continuum and categorical beliefs	4	Schizophrenia	Rating	Likert (1–4)
11	Helmus et al. (2019)	Intervention with follow-up (paper–pencil)	t1: *n* = 202; mean age = 45.5 years; 34.7% male t2: *n* = 131; mean age = 45.2 years; 35.1% male	Mental health care professionals	Netherlands	CBQ	16	Schizophrenia	Rating	Likert (1–6)
12	Makowski et al. (2016)	Cross-sectional (online)	*n* = 2006; mean age = 47.5 years; 47.9% male	General population	Germany	Belief in a continuum of symptom experience	1	Depression; schizophrenia	Vignette	Likert (1–4)
13	Makowski et al. (2021)	Cross-sectional (telephone survey)	*n* = 1009; 18 to ≥ 65 years; 49% male	General population	Germany	Continuity beliefs; fundamental differences	4	Depression	Vignette	Likert (1–4)
14	Morris et al. (2020)	Cross-sectional (online)	*n* = 597; mean age = 37.21 years; 52.9% male	General population	United Kingdom	PDBS	5	Alcohol use disorder	Vignette	Likert (1–5)
15	Norman et al. (2008)	Cross-sectional (paper–pencil)	*n* = 200; mean age = 21.5 years; 45% male	Undergraduate students	Canada	Belief in a continuum of symptom experience	3	Depression; schizophrenia	Vignette	Likert (1–5)
16	Norman et al. (2010)	Repeated cross-sectional (paper–pencil)	Study 1 *n* = 200; mean age = 21.5 years; 45% male Study 2 *n* = 103; mean age = 55.7 years; 50.5% male	Study 1: undergraduate students Study 2: community service club members	Canada	Belief in a continuum of symptom experience	3	Depression; schizophrenia	Vignette	Likert (1–5)
17	Paulus et al. (2015)	Cross-sectional (online)	*n* = 270; mean age = 26.8 years; 19.6% male	Undergraduate students	USA	Belief in a continuum of symptom experience	1	Depression; social anxiety disorder; generalized anxiety disorder	Vignette	Severity rating (0–8)
18	Schlier et al. (2016)	Repeated cross-sectional (online)	Study 1: *n* = 95; mean age = 26.37; 50.5% male; Study 2: *n* = 363; mean age = 27.4 years; 34.7% male Study 3: *n* = 229; mean age = 37.3 years; 38.4% male	Study 1: online sample Study 2: online sample Study 3: general population	Germany	CBQ; CBQ-R	16; 14	Schizophrenia	Rating	Likert (1–7)
19	Schlier et al. (2019)	Online experiment	*n* = 137; mean age = 27.8 years; 28.5% male	Undergraduate students + online sample	Germany	Perceived similarity	4	Depression; schizophrenia	Vignette	Likert (1–6)
20	Schomerus et al. (2013)	Cross-sectional (face to face, paper–pencil)	*n* = 3642; > 18 years; 45.6% male	General population (representative)	Germany	Belief in a continuum of symptom experience	1	Depression; schizophrenia; alcohol use disorder	Vignette	Likert (1–5)
21	Schomerus et al. (2015)	Repeated cross-sectional (online)	Study 1 *n* = 598; Study 2 *n* = 806; > 15 years	General population (representative)	Germany	Belief in a continuum of symptom experience	1	Depression; schizophrenia	Vignette	Likert (1–5)
22	Schomerus et al. (2016)	Online experiment	*n* = 1679; > 15 years; 49% male	General population (representative)	Germany	Continuity beliefs; fundamental differences	4	Depression; schizophrenia	Vignette	Likert (1–5)
23	Schomerus et al. (2022)	Repeated cross-sectional (face-to-face)	Study 1: *n* = 2455; 18 to ≥ 61 years; 45.6% male; Study 2: *n* = 3042; 18 to ≥ 61 years; 47.2% male;	General population (representative)	Germany	Belief in a continuum of symptom experience	1	Depression; schizophrenia	Vignette	Likert (1–5)
24	Seow et al. (2017)	Cross-sectional (online)	*n* = 500; 16.6% male	Undergraduate students	Singapore	Belief in a continuum of symptom experience	1	Depression; schizophrenia; alcohol use disorder; dementia, OCD	Vignette	Likert (1–5)
25	Speerforck et al. (2019)	Cross-sectional (telephone survey)	*n* = 1008; > 18 years	General population (representative)	Germany	Belief in a continuum of symptom experience	1	ADHD	Vignette	Likert (1–5)
26	Subrahamian et al. (2017)	Cross-sectional (online)	*n* = 3006; 18–65 years; 50.9% male	General population (representative)	Singapore	Belief in a continuum of symptom experience	1	Depression; schizophrenia; alcohol use disorder; dementia, OCD	Vignette	Likert (1–5)
27	Thibodeau & Peterson (2018)	Laboratory experiment (paper–pencil)	*n* = 135; mean age = 18.7 years; 23.0% male	Undergraduate students	USA	Endorsement of Continuum/Categorical beliefs	4	Schizophrenia	Vignette	Likert (1–4)
28	Thibodeau (2017)	Online experiment	*n* = 308; mean age = 33.8 years; 54.9% male	MTurk sample	USA	Continuum and categorical beliefs	1	Schizophrenia	Vignette	Likert (1–5)
29	Thibodeau (2020)	Online experiment	*n* = 654; mean age = 29.6 years; 39.1% male	MTurk sample	USA	Endorsement of Continuum/Categorical beliefs	4	Depression	Vignette	Likert (1–4)
30	Thibodeau, Shanks et al. (2018)	Laboratory experiment (paper–pencil)	*n* = 69; mean age = 18.7 years; 17.4% male	Undergraduate students	USA	Endorsement of Continuum/Categorical beliefs	4	Schizophrenia	Vignette	Likert (1–4)
31	Thoerel et al. (2022)	Online experiment	*n* = 725; mean age = 32.03 years; 31.3% male	General population	Germany	General concept of mental health	8	Eating disorders (anorexia nervosa, bulimia nervosa, binge eating disorder)	Vignette	Likert (1–5)
32	Violeau et al. (2020)	Online experiment	*n* = 565; mean age = 26.0 years; 34.5% male;	General population (mainly undergraduate students)	France	QBCS (adapted from the CBQ)	4	Schizophrenia	Rating	Likert (1–7)
33	von dem Knesebeck et al. (2015)	Repeated cross-sectional (telephone survey)	Study 1: *n* = 650; > 18 years; 47.9% male Study 2: *n *= 601; > 18 years; 48.1% male	General population (representative)	Germany	Belief in a continuum of symptom experience	1	Depression	Vignette	Likert (1–5)
34	Wiesjahn et al. (2014)	Cross-sectional (online)	*n* = 120; mean age = 31.5 years; 21.7% male	General population	Germany	CBQ	16	Schizophrenia	Rating	Likert (1–6)
35	Wiesjahn et al. (2016)	Online experiment	*n* = 1189; mean age = 30.98 years; 32.3% male	General population	Germany	CBQ	16	Schizophrenia	Rating	Likert (1–6)

*Notes*. All measures were self-report measures and one-dimensional; *CBQ *Continuum Beliefs Questionnaire, *PDBS *Problem Drinking Belief Scale, *OCD* Obsessive Compulsive Disorder, *MTurk* Amazon Mechanical Turk, a crowdsourcing platform, *QBCS *Questionnaire of Belief in a Continuum in Schizophrenia

Overall, most studies focused on schizophrenia (*n* = 23) or depression (*n* = 20), followed by alcohol use disorder or addiction (*n* = 5), OCD (*n* = 3), and dementia (*n* = 2). One study each measured continuum beliefs regarding ADHD, social anxiety disorder/generalized anxiety disorder, eating disorder, and bipolar disorder. To elicit continuum beliefs, 27 out of 35 studies utilized vignettes, sometimes personalized with names and/or gender. These vignettes consisted of short descriptions of either a person with a specific disorder or typical symptoms of said disorder based on its diagnostic criteria according to DSM-IV or ICD-10. Eight studies used a rating scale, for instance the Continuum Beliefs Questionnaire (CBQ), that measures continuum beliefs independent of a vignette [52, 58, 64, 65]. All studies, except one [57], used four-point to seven-point Likert scales as response measures (i.e., agreement with statements about a person, symptoms or a condition). The remaining study [57] asked participants to rate the severity of presented vignettes on a scale from 0 to 8 and provided a hint that experts perceived a rating above four as clinically relevant.

Eighteen studies investigated general population samples, with nine explicitly mentioning representativeness of their sample (e.g., stratified sampling and weighted analysis). However, studies rarely mentioned how representativeness was achieved, for instance, via quota sampling or probability sampling; therefore, this information is not included in Table 1. Seven studies examined (undergraduate) students, seven used Amazon Mechanical Turk (MTurk) samples, and three investigated adolescents [51, 70], or mental health professionals [52].

### Content of continuum belief measures

**Fig. 2:**
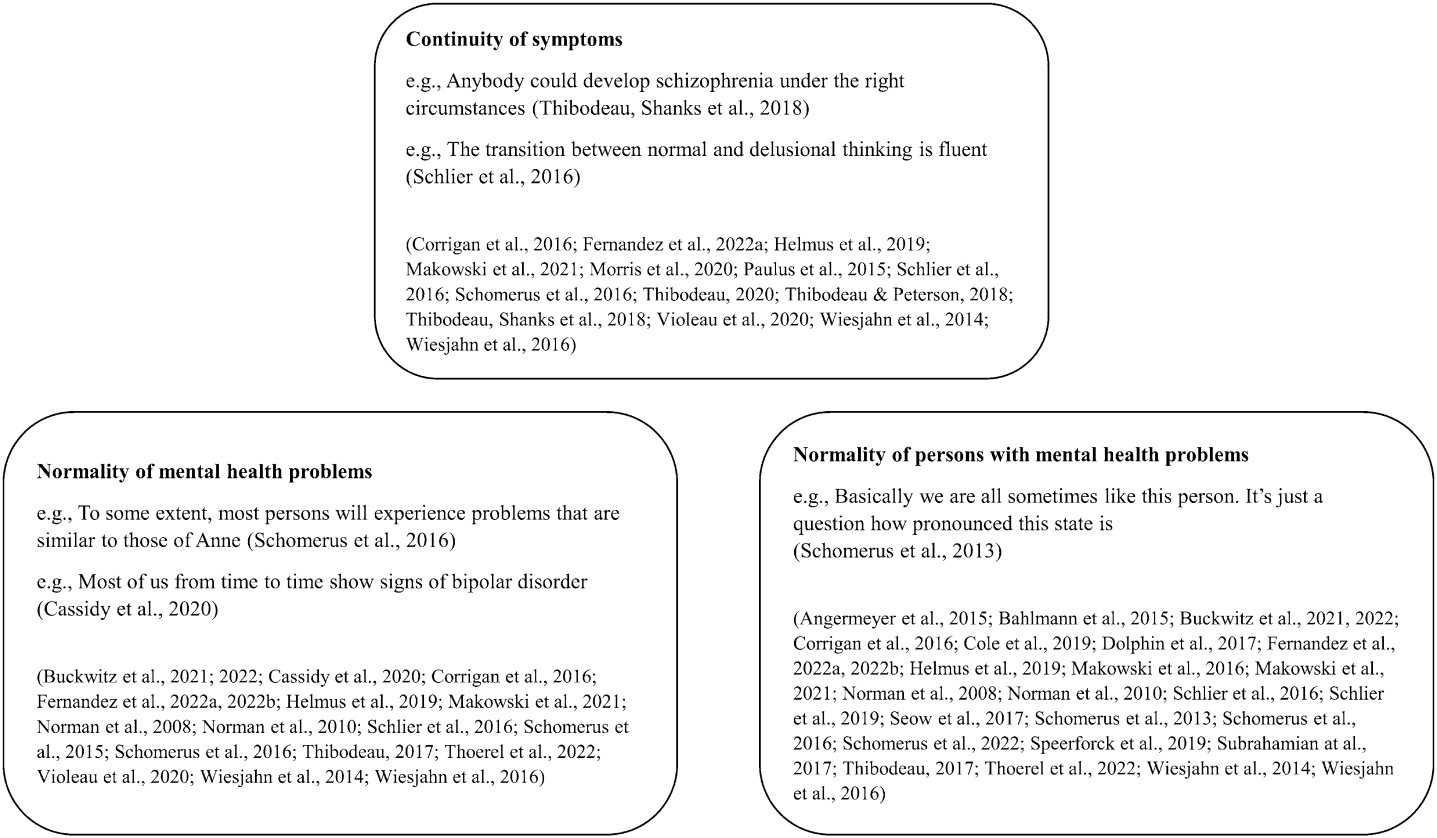
Three measured core aspects of continuum beliefs

Eleven different measures were used across studies, and all were analyzes as one-dimensional measures. They ranged from single-item measures for general continuum beliefs [e.g., “Basically we are all sometimes like this person. It’s just a question how pronounced this state is.“; 60] to illness-specific scales with sixteen items [schizophrenia; 64], four items [schizophrenia; 81] and five items [problem drinking/addiction; 54]. Three measures, namely, Continuum Beliefs Questionnaire (CBQ), Questionnaire of Belief in a Continuum of Schizophrenia (QBCS), and Problem Drinking Belief Scale (PDBS), received distinct labels, and other measures did not, despite being used in multiple studies. The single-item measure by Schomerus et al. [60], for instance, was used or adapted by ten of the included studies [22, 48, 49, 51, 53, 59, 61–63, 74], one of which [49] performed additional analyses with the same data set as the original study [60]. Two studies [66, 67] also referred to one data set. Most measures aim to assess beliefs in a continuum of symptom experience (see Table 1). However, a closer look at the items used in these measures reveals three different aspects of continuum beliefs, namely, (1) continuity of symptoms [e.g., "The transition between normal and delusional thinking is fluent"; 58], (2) normality of mental health problems [e.g., “To some extent, most persons will experience problems that are similar to those of Anne”; 59], and (3) normality of persons with mental health problems [e.g., “Basically, we are all sometimes like this person”; 60]. Conceptually, the first continuum closely resembles the continuous understanding of mental health and mental illness, as expressed, for instance, in the dimensional operationalization of mental disorders in the DSM 5 or the psychosis continuum [1, 4]. The second and third continua rely either on a personal experience of symptoms or the identification with a person with mental illness (i.e., a vignette). Both refer to a norm of inclusivity (e.g., we are all like this person, most people experience these symptoms) rather than a continuum of symptoms to represent mental illness. They are not necessarily linked to the nosological concept of an illness but rather to its phenotype and prevalence (second continuum) and the perceived similarity or lack of perceived differentness regarding the vignette (third continuum). Perceived differentness is often used as an indicator of stigmatizing attitudes, since it depicts the differentiation between us and them, which is a core process of stigmatization [8]. The identified continua, exemplary items, and the assigned studies are presented in Fig. 2.

While most measures focus on only one or two aspects of continuum beliefs, two measures represent all three aspects of continuum beliefs, namely, the scale developed by Schomerus et al. [59] and the CBQ [64]. The former is generic and vignette-based, the latter was specifically developed as a rating scale for continuum beliefs regarding schizophrenia. Despite their inclusion of all three aspects, both measures were analyzed as one-dimensional scales, and the conceptual differences between continua were not explored any further. In addition, no study has empirically compared different measures or operationalizations of continuum beliefs.

In the next step, we examined methodological quality, psychometric properties, and utility (i.e., readability, cultural translation, respondent burden) of continuum belief measures across studies. Categories and ratings were based on previous research [46, 47], and rated independently by the first and third author. Differences were discussed and resolved with the second author (see Table 2).

**Table 2:** Psychometric properties of continuum belief measures in the included studies (*n* = 35)

No.	Study	Readability	Cultural translation	Respondent burden	Content validity	Criterion validity	Construct validity	Internal consistency	Test–retest reliability	Responsiveness
1	Angermeyer et al. (2015)	+ +	+ +	+ +	+		+			
2	Bahlmann et al. (2015)	+ +		+ +	+ +					
3	Buckwitz et al. (2021)	+ +		+ +	+	+ +	+	+		+ +
4	Buckwitz et al. (2022)	+ +		+ +	+	+ +	+	+		+ +
5	Cassidy et al. (2020)	+	+	+ +			+	+ +		+ +
6	Cole et al. (2019)	+ +		+ +	+ +		-			+ +
7	Corrigan et al. (2016)	+		+ +	+			+		+ +
8	Dolphin et al. (2017)	+ +		+ +	+ +					+ +
9	Fernandez et al. (2022a)	+ +		+ +	+	+ +	+	-		
10	Fernandez et al. (2022b)	+ +		+ +	+			-		+ +
11	Helmus et al. (2019)	+ +	+ +	+ +	+			+ +		
12	Makowski et al. (2016)	+ +	+ +	+ +	+	+ +	+			
13	Makowski et al. (2021)	+ +		+ +	+			-		
14	Morris et al. (2020)			+ +			+	-		
15	Norman et al. (2008)	+ +	+	+ +	+ +	+ +	+ +		+ +	
16	Norman et al. (2010)	+ +	+	+ +	+ +			+	+ +	
17	Paulus et al. (2015)	+ +		+ +	+					
18	Schlier et al. (2016)	+ +	+	+ +	+ +	+ +	+ +	+		
19	Schlier et al. (2019)	+ +	+	+ +				+		+ +
20	Schomerus et al. (2013)	+ +	+ +	+ +	+		+ +			
21	Schomerus et al. (2015)	+ +	+	+ +						
22	Schomerus et al. (2016)	+ +	+ +	+ +	+	+ +	+ +			+ +
23	Schomerus et al. (2022)	+ +		+ +	+	+ +	+ +			
24	Seow et al. (2017)	+ +		+ +	+	+ +		+ +		
25	Speerforck et al. (2019)	+ +	+ +	+ +	+ +	+ +	+ +			
26	Subrahamian et al. (2017)	+ +	+ +	+ +	+	+ +		+		
27	Thibodeau & Peterson (2018)	+		+ +		+ +	+ +			+ +
28	Thibodeau (2017)	+ +		+ +	+ +	+ +				+ +
29	Thibodeau (2020)	+	+	+ +	+	+ +	+ +			+ +
30	Thibodeau, Shanks et al. (2018)	+		+ +	+	+ +	+			+ +
31	Thoerel et al. (2022)	+ +	+ +	+ +	+			+		+ +
32	Violeau et al. (2020)	+ +	+	+ +	+		+			-
33	von dem Knesebeck et al. (2015)	+ +	+ +	+ +	+					
34	Wiesjahn et al. (2014)	+ +	+ +	+ +	+ +	+ +	+	+		
35	Wiesjahn et al. (2016)	+ +		+ +	+ +	+ +	+ +	+		+ +

*Notes*. Empty cells mean that no information was available/reported; Readability: + items available but lengthy; +  + items available, short, and comprehensive; Cultural translation: + only available in English; +  + available in English and/or language(s) of the target population; Respondent burden: + over 60 items; +  + under 60 items; Content validity: + theoretical foundation; +  + theoretical foundation, and experts consulted; Criterion validity: +  + Correlation coefficient with external criteria calculated (e.g., other measures of continuum or categorical beliefs); Construct validity:—non-significant correlations with related (i.e., concurrent validity) and/or non-related constructs (i.e., discriminant validity); + low correlations with related and/or non-related constructs; +  + moderate to strong correlations with related and/or non-related constructs; Internal consistency:—mean Cronbach’s alpha below .70; + mean Cronbach’s alpha between .70 and .80; +  + mean Cronbach’s alpha at least equal to .80; Test–retest reliability: +  + significant test–retest correlation across at least two different timepoints; Responsiveness:—unsuccessful manipulation check; +  + successful manipulation check (i.e., significant changes in continuum beliefs following a continuum belief intervention)

Overall, most studies pointed to good readability, content validity and low respondent burden. Criterion validity was also very positive for most measures across studies. Cultural translation of some measures was proven, for instance, the adapted measure of Schomerus et al. [60]. All measures were comparably short (1–16 items), which makes them highly economical and efficient. Content validity and criterion validity were also high for most studies, since measures were based on theoretical considerations, pretested and validated, for example, via manipulation tests, and expert consultations. Construct validity was mostly tested as discriminant validity resulting in either low or negative correlations between continuum beliefs and stigmatizing attitudes in most studies except for one study on OCD [50]. Fewer studies reported (satisfactory) internal consistency (e.g., Cronbach’s alpha > 0.7), test–retest reliability was reported in two studies [55, 56]. Floor or ceiling effects were not explicitly reported in any of the included studies. Since all measures were self-reports and few studies examined continuum beliefs at multiple timepoints to calculate test–retest reliability, intra-rater reliability as well as inter-rater reliability were also not reported. Responsiveness was very good, as many studies used experimental designs and manipulation checks to measure changes in continuum beliefs following continuum belief interventions. None of the studies reported known-groups validity (e.g., based on gender, age or type of disorder) regarding continuum beliefs measures. As a summary, an overview of measures is provided in Table 3.

**Table 3:** Overview of eleven measures of continuum beliefs (plus a revised version of the Continuum Beliefs Questionnaire) including their origin, number of items, assessment method, and the dimensions of continuum reflected with each measure as well as examined disorders

Measure	Origin	No. of items	Method	Country	Type of continuum	Examined disorders
Belief in a continuum of symptom experience	Schomerus et al. (2013)	1	Vignette	France; Germany; Singapore; Ireland	Normality of persons with mental health problems	Depression; schizophrenia; alcohol use disorder; dementia, obsessive–compulsive disorder; attention deficit hyperactivity disorder
Continuity beliefs	Schomerus et al. (2016)	4	Vignette	Germany	Continuity of symptoms Normality of mental health problems Normality of persons with mental health problems	Depression; schizophrenia
Continuum beliefs	Thibodeau (2017)	1	Vignette	USA	Normality of mental health problems	Schizophrenia; obsessive–compulsive disorder
Endorsement of continuum beliefs	Thibodeau, Shanks et al. (2018)	4	Vignette	USA	Continuity of symptoms	Schizophrenia
Continuum Beliefs Questionnaire	Wiesjahn et al. (2014)	16	Rating	Germany; USA; Netherlands	Continuity of symptoms Normality of mental health problems Normality of persons with mental health problems	Schizophrenia
Continuum Beliefs Questionnaire-revised	Schlier et al. (2016)	14; 16	Rating	Germany	Continuity of symptoms Normality of mental health problems Normality of persons with mental health problems	Schizophrenia
Belief in a continuum of symptom experience	Norman et al. (2008)	3; 4	Vignette	Canada; USA	Normality of mental health problems Normality of persons with mental health problems	Depression; schizophrenia; bipolar disorder
Perceived similarity	Schlier et al. (2019)	4	Vignette	Germany	Normality of persons with mental health problems	Depression; schizophrenia
Belief in a continuum of symptom experience	Paulus et al. (2015)	1	Vignette	USA	Continuity of symptoms	Depression; social anxiety disorder; generalized anxiety disorder
Problem Drinking Belief Scale	Morris et al. (2020)	5	Vignette	USA	Continuity of symptoms	Alcohol use disorder
General concept of mental health	Thoerel et al. (2022)	8	Vignette	Germany	Normality of mental health problems Normality of persons with mental health problems	Eating disorders
Questionnaire of Belief in a Continuum in Schizophrenia	Violeau et al. (2020)	4	Rating	France	Continuity of symptoms Normality of mental health problems	Schizophrenia

## Discussion

This systematic review summarizes and evaluates measures of continuum beliefs of mental illness. The search identified eleven different measures that ranged from single items to multi-item scales. Most scales were generic, but some were developed for specific disorders (i.e., schizophrenia, alcohol use disorder). The measures seem to have high objectivity, since the instructions are clear, readability is high, and they are easy to implement. Most measures also have high validity due to their theory-based development, pretests, and psychometric testing (see Table 2). Yet, other psychometric properties such as reliability (e.g., test–retest reliability) as well as clinical utility have rarely been investigated beyond initial piloting studies and reports of internal consistency. Thus, more extensive psychometric studies are needed to test factorial validity and measurement invariance, test–retest reliability, and cross-cultural validity. The latter is particularly important given cross-cultural differences in conceptualizing mental disorders that might influence continuum beliefs [e.g., 82, 83].

Although some measures have been adapted to different European, American, and Asian contexts [60], further comparative cross-cultural research is encouraged. Moreover, the development, harmonization, and monitoring of continuum belief measures should be connected to novel developments in describing and diagnosing mental disorders. Paradigms such as HiTOP [84] aim to provide an overarching hierarchy of psychopathology that pays respect to cross-cultural differences and focuses on phenotypical similarities, thus continuum belief measures could be developed and extended in tandem.

The continuum belief measures were mostly implemented in general population samples which supports their feasibility and applicability for epidemiological research. Epidemiological mental health cohorts, for instance, could incorporate these measures to assess not only stigmatizing attitudes but also continuum beliefs. Similarly, anti-stigma campaigns could include continuum belief measures to measure efficacy concerning public health impact, due to mostly robust negative associations between continuum beliefs and stigmatizing attitudes [36]. However, in some studies [e.g., 50, 81], this association was not significant; the continuum belief intervention even lead to an increase in self-stigma (i.e., *being weird/unpredictable is typical of me*) in one study [81]. The authors [81] argue that this type of non-threatening self-stigma (e.g., weird as opposed to dangerous) is an expression of increasing perceived similarities to the target group thus strengthening shared social identity. However, it is unclear how this affects persons with more severe symptoms and perceived similarity with more threatening attributes (e.g., dangerous). Potentially, continuum belief interventions could exacerbate group differences in samples with more severe symptoms, because vignettes of disorders with mild to moderate severity (as used in continuum belief measures) highlight the discrepancy between normal functioning and their personal experience. For example, in a study by Thibodeau and Peterson [78], the continuum belief intervention increased fear. This conclusion is merely hypothetical, though because of a lack of studies with a varying severity of symptoms and mental disorders.

Overall, more studies with clinical samples and mental health professionals are needed to assess clinical utility and practicability. One study with persons with at risk alcohol use [54] provided tentative evidence that promoting continuum beliefs might increase problem recognition. Problem recognition is an important predictor of treatment motivation following the transtheoretical model of health behavior change [85, 86], and it can lead to lower drop-out rates, which is very promising for this field [87]. Therefore, the function of continuum beliefs in treatment processes needs to be studied more closely. This is also true for more diverse populations (e.g., children and adolescents, older adults). One study with adolescents showed good psychometric properties of continuum belief measures [51], but more research is necessary to confirm these findings. Since several studies used random online samples (gathered via MTurk), their results should also be interpreted with caution when thinking about adapting scales to applied contexts, since there is an ongoing debate about data quality and validity of MTurk data and similar online panels and services compared to pragmatic, and community samples [88–90]. Hence, multi-group comparisons of samples from different providers and sources are recommended.

Furthermore, the conceptualization of continuum beliefs needs to be examined. The CBQ, the PDBS, and the QBCS were developed for specific disorders, which is why they can refer to disorder-specific symptoms without including vignettes or descriptions of mental disorders as a frame of reference. Consequently, other studies did not need to adapt or pretest additional materials. These scales could also directly describe a disorder-specific continuum of symptoms (e.g., the psychosis continuum; [4]) as an indicator of mental stress leading to mental illness, which is in line with the approach of normalization proposed by Angermeyer und Schulze [21]. Vignette-based studies with more generic scales, on the other hand, were more flexible and allow direct comparisons of beliefs regarding different disorders—which lends credibility to the idea of an underlying concept of continuity or dimension of mental health and illness. This way of thinking corresponds to current positive psychological approaches, such as the dual continuum model of mental health [43, 44], and the HiTOP model with its focus on phenotypes rather than diagnostic labels or categories [84].

This more generic approach, however, also requires validated vignettes to assess continuum beliefs. This is challenging for multiple reasons: First, the included studies used different vignettes which could have biased the results. Second, most studies controlled for confounding influences by either presenting no gender or name or randomizing gendered vignettes. However, these vignettes still required participants to imagine the person and their symptoms, which requires sufficient perceived realism of each vignette and consensus regarding the described experience (e.g., of a depressive episode) [91]. Therefore, future research should compare continuum beliefs across different vignettes. Third, other aspects such as age or ethnicity of the presented or imagined person were not controlled and might have additional influence on continuum beliefs [92]. Hence, future studies should examine the differential impact of different disorder-specific vignettes on multiple measures of continuum beliefs. These vignettes could also be tested or constructed based on population assessments, similar to the measure of Paulus et al. [57] In their study, they asked participants to rate the severity of different symptoms and behaviors ranging from healthy to clinically relevant. While this is closely connected to a diagnostic approach (e.g., in psychotherapeutic training), it also provides the opportunity to customize (sub-)clinical vignettes of specific disorders concerning type and intensity of symptoms and assess subsequent ratings to examine the extent of continuum beliefs. In this sense, future research could build upon scale-based measures, such as the CBQ that requires similar assessments (e.g., regarding hallucinations) via Likert scales.

Finally, different operationalizations of continuum beliefs are also a promising avenue for future research, similar to the area of health literacy, where multiple objective tests and subjective self-reports are state of the art [93, 94]. While the identified measures captured between one and three aspects of the continuum (see Fig. 2), certain aspects were rarely examined, for example, the categorical v continuous conceptualization of mental illness [2, 3]. Items measuring this nosological concept were included in the development of the CBQ, but they were eventually excluded from the final measure [64]. It might be beneficial to compare measures of such conceptual beliefs with continuum beliefs measures, and compare multiple measures of continuum beliefs, to assess similarities and differences and examine their responsiveness in future interventional studies. Nevertheless, it should also be added that a more conceptual measure of continuum beliefs requires a more abstract assessment of nosological concepts of illness and health, which might be rather difficult for laypersons, meaning population samples without previous education about this issue.

In sum, when choosing a measure of continuum beliefs, a researcher needs to think about the population (e.g., a sample with clinical depression vis-à-vis a healthy population sample), the context (e.g., disorder-specific versus transdiagnostic assessments), the method (e.g., rating scales versus vignettes), and the overall aim of the study (e.g., comparing attitudes across groups or disorders versus examining predictive utility or validity of continuum beliefs). In an epidemiological study of depression-related attitudes in the population, a disorder-specific measure using vignettes might be most appropriate, whereas a comparative study of continuum beliefs across different disorders might benefit from a short, generic measure that has a low respondent burden and allows for transdiagnostic comparisons. While our review shows that some types of measures have received more attention than others so far, the usefulness and merit of each measure strongly depends on the context of investigation. This review provides a framework for decision-making and further research in continuum beliefs of mental illness.

The review is not without limitations. The search was limited to three data bases, and preregistered search criteria (e.g., regarding search terms, language) as well as peer-reviewed literature, which might have neglected grey literature and other studies that could not be identified by the initial search. The review focused on continuum beliefs of mental illness, while previous literature defined different continua (e.g., continuum of care, dual continua model) that might be associated with continuum beliefs. For instance, the continuum of care assumes different needs and responsibilities for different stages of an illness, such as prevention, acute treatment, or recovery [95]. These stages are associated with different levels of severity of an illness, which might serve as a reference for assessing continuum beliefs. Similarly, the dual continua model assumes parallel continua of mental well-being and mental health/illness. It is unclear how different constellations of well-being and mental health (e.g., flourishing) are associated with continuum beliefs. The study used established ratings of methodological quality and it reported results in accordance with the PRISMA statement, but it did not examine risk of bias or use different rating systems of measures. This could be the focus of future work. Despite its weaknesses, however, this review identified several measurement instruments of continuum beliefs with applications in multiple cultural contexts, and initial evidence of good validity, and applicability in general population samples. Hence, the potential of continuum beliefs regarding public mental health and the economic modes of assessment are quite promising.

## Data Availability

Data sharing is not applicable to this article as no new data were created or analyzed in this study.
